# An innovative way of thinking nuclear waste management – Neutron physics of a reactor directly operating on SNF

**DOI:** 10.1371/journal.pone.0180703

**Published:** 2017-07-27

**Authors:** Bruno Merk, Dzianis Litskevich, Mark Bankhead, Richard J. Taylor

**Affiliations:** 1 University of Liverpool, School of Engineering, Liverpool, United Kingdom; 2 National Nuclear Laboratory, Chadwick House, Warrington, United Kingdom; 3 University of Manchester, School of Mechanical Aerospace & Civil Eng., Manchester, United Kingdom; Los Alamos National Laboratory, UNITED STATES

## Abstract

A solution for the nuclear waste problem is the key challenge for an extensive use of nuclear reactors as a major carbon free, sustainable, and applied highly reliable energy source. Partitioning and Transmutation (P&T) promises a solution for improved waste management. Current strategies rely on systems designed in the 60’s for the massive production of plutonium. We propose an innovative strategic development plan based on invention and innovation described with the concept of developments in s-curves identifying the current boundary conditions, and the evolvable objectives. This leads to the ultimate, universal vision for energy production characterized by minimal use of resources and production of waste, while being economically affordable and safe, secure and reliable in operation. This vision is transformed into a mission for a disruptive development of the future nuclear energy system operated by burning of existing spent nuclear fuel (SNF) without prior reprocessing. This highly innovative approach fulfils the sustainability goals and creates new options for P&T. A proof on the feasibility from neutronic point of view is given demonstrating sufficient breeding of fissile material from the inserted SNF. The system does neither require new resources nor produce additional waste, thus it provides a highly sustainable option for a future nuclear system fulfilling the requests of P&T as side effect. In addition, this nuclear system provides enhanced resistance against misuse of Pu and a significantly reduced fuel cycle. However, the new system requires a demand driven rethinking of the separation process to be efficient.

## Introduction

The nuclear waste problem is in the public recognition one of the key problems to be solved to provide the basis for an extensive use of nuclear reactors reactors as a major carbon free, sustainable, and applied highly reliable energy source. The technology of Partitioning and Transmutation (P&T) has the potential to provide a technological solution for improved nuclear waste management due the significantly reduced long term challenge on a possible repository site [1]. The key components for successful installation of a P&T cycle are the separation technology for the transuranic isotopes, the pellet/solid fuel production, and a fast reactor for burning the separated transuranic isotopes and these stages have to be operated in a multi cycle mode. Most of the currently envisaged components are based on existing technologies like aqueous reprocessing and sodium cooled fast reactor technology. This is the current state of the art of the proposed P&T technology. To develop a disruptive innovation to overcome the hurdles of the current status a more generic view will be put onto the technological development by analysing the strategic development options.

Strategic development is the key to any long term success of industrial innovation. During the development of any new technology important decisions have to be made on the way which are typically based on current external drivers like political, economic or technical boundary conditions. However, these drivers often undergo significant changes during the long term development of a sophisticated technology. This leads to a change in the objectives and the final outcome differs very often significantly from the objectives given at begin of the development. Especially, when developing a new technology with significantly differing demand these historic decisions could be cumbersome for the progress of a future technology. In the 1960s, Everett Rogers described the development of innovation through S-curves. He fixed these thoughts in the theory of diffusion of innovations [2]. Rogers’ argues that the application of a technology to a market follows these S-curves. The economist Fredmund Malik extended this thinking and titled it a “Symphony of S-curves: Seeing the Future Clearly” in his book on strategy development [3] to motivate the people to leave the beaten tracks when it is requested by changed boundary conditions. He describes the development not with a single s-curve anymore but by a finite number of successive curves. He invented the idea of changing from one s-curve to another more advanced one when radical innovation is required by the changed market surrounding.

In the first part of this publication, the development concept of Rogers and Malik is applied to analyze the current development of nuclear reactor systems in general. The historic boundary conditions are reviewed against the current boundary conditions with special regards to sustainability and P&T. In the second part, the results of the strategic development discussion including the updated boundary conditions will be used to define a reactor system which is as close as possible to the requests of a wide spread future electric energy production by nuclear power and the feasibility of the neutron physical challenge is demonstrated. The requests of the P&T technology will provide here one of the major keys since it is in our view a major request to create the basis for the long term success and acceptance of nuclear reactors in the future. Applying innovative technologies will be the basis for nuclear to act as a major contributor providing reliable carbon free, sustainable electric energy.

## The development of nuclear technologies

The general development of nuclear reactors started in 1942, with the “Chicago Pile 1” which was the world's first nuclear reactor, built by Nobel Prize winner Enrico Fermi [4]. During the first years of the development of nuclear technologies, the purpose of a nuclear reactor was based around three major objectives [5]:

1. Demonstration of a self-sustained chain reaction and its potential application
2. Production of material for military purposes and/or commercial or medical applications
3. Demonstrating energy generation using the chain reaction, either as electricity, or heat

**Fig 1:**
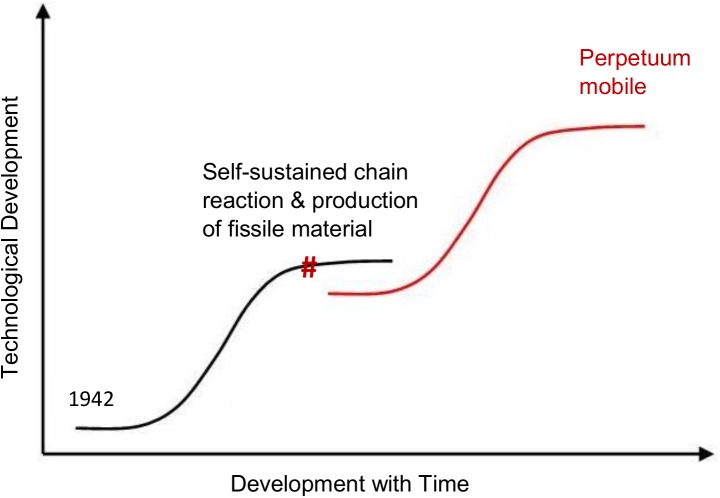
The nuclear reactor development in S-curves.

During the last centuries the main focus of nuclear reactors operation has changed significantly to the generation of reliable carbon free energy in a sustainable way, literally an advancement of objective 3 which forms the objective of the newly introduced s-curve (red curve in Fig 1) headed by the vision of all kinds of energy production. Objective 1 has been extensively proven and objective 2 is secured using specialised technologies. Instead of these two objectives, a new objective has developed with the growing use of nuclear reactors, the request for a solution of the accumulation of spent nuclear fuel which is declared as nuclear waste.

The current state of nuclear reactors, the light water reactor (LWR) technology is still essentially based on technology developed to power nuclear submarines, even if it has undergone a successive evolution to the Generation III/III+ reactors. Projecting the current light water reactor development status onto a s-curve of development (black curve in Fig 1), the Gen III/III+ reactors appear in the almost asymptotic top end of the curve, see red cross in Fig 1. This status is characterized by a highly competitive market, almost converged solutions from different providers, and small evolutionary development steps to complete the optimization. LWRs are to be seen as almost ideal solution under the currently given boundary conditions. However, how does this look like for the P&T relevant separation and fast reactor technology?

On the one hand, the current fast reactor development and the surrounding fuel cycle is driven by the Generation IV (GenIV) goals [6]

- Sustainability
- Safety and reliability
- Economics
- Proliferation resistance and physical protection

which build the bridge between the current S-curve and a possible future one (red curve in Fig 1). On the other hand, the technology of most of the fast reactors proposed in Gen IV goes back to the early stages of reactor development, to the time of the “atoms for peace” speech and the foundation of the IAEA [7]. “Seen from the historic point of view given above almost all [Generation IV] systems are based on the very early developments of nuclear reactors when the development objective has been formed with completely different boundary conditions and requests as we have it today–producing nuclear materials and powering submarines versus wide spread sustainable nuclear power production.”[5] This is not only reflected by the first generation of fast reactors [8] developed to produce high quality fissile material for nuclear weapons, but also by the PUREX (Plutonium Uranium Redox EXtraction) process for the separation of this high quality fissile material [9].

### Demand driven research

Most of the important decisions during the development of nuclear power and of fast reactor technology, with the link to the specific fuel cycle, have been made on the historic demand cited above to develop the ideal system for these given demand. In our view, it is time to revisit these old decisions posing several questions “Would these decisions be taken the same way under today’s boundary conditions? Would we take the branch-off once more into the same direction, even if we are travelling to another destination?” The community should question their decision makers permanently; “Are we on the right way to the destinations of the future?”

Some very interesting examples can be found for these historic decisions at important branch off points. One of the most prominent could be the decision between the molten salt breeder reactor developed at the Oak Ridge National Laboratory and the sodium cooled fast reactor developed at the Argonne National Laboratory, in the early seventies [9]. The sodium cooled fast reactor with solid fuel is advantageous for the production of high quality fissile material. Thus, supporting this system was definitely the right decision to fulfil the demand of the 70ies but will this decision be advantageous for the wide spread sustainable nuclear power production and the transmutation of nuclear waste, too? Another point is the decision for the Plutonium Uranium Redox EXtraction (PUREX) process. What are we heading for in reprocessing spent nuclear fuel? We try to take out what we need on the level of the 70ies objective, the Pu like the name of the process defines. Thus the combination of the sodium cooled fast breeder reactor and the PUREX process was ideal for the demand at the time of the development–producing and separating high quality fissile material. However, the objective of today is the highly sustainable energy production in nuclear reactors including P&T. To fulfil the today’s demand with these processes, we have to achieve extremely high recovery factors to avoid plutonium being forwarded to the final disposal. For P&T, the key chemical step is the partitioning of the spent fuel to get access to the materials which are planned to be transmuted. The initial step is still based on the PUREX process, but with added downstream process steps like the DIAMEX to separate lanthanides, Americium and Curium, and one of the SANEX processes to separate Americium and Curium form the lanthanides [10]. To avoid possible proliferation concerns which arise from the pure plutonium fraction in the PUREX process, new separation processes like COEX or GANEX are under development for future application [10, 11]. In addition there have nonaqueous processes developed which rely on processes in molten chloride salts [11]. In the view of demand driven research we have to ask ourselves,”why do we not only take out what disturbs/prevents the reactor operation instead of separating fissile materials?” Maybe we have to invent new processes for this purpose. We should ask “Can we fulfil the demand in easier and less complex processes?” Do we really need this extremely high purity or is it just a drawback of the processes we have chosen in past? Do we need this extremely complex fuel cycle for a future technology like P&T or could we design a reactor for sustainable energy production and a significantly reduced process scheme around? Can we adopt our reactor system to reduce the requests for the other processes e. g. for reprocessing, mining, enrichment…?

Yes we can and in our view, but we have to provide disruptive innovation to make nuclear technologies attractive for the future! Only, if we are willing to leave the evolutionary development to disrupt the chain formed by the direction/way we have decided for in the 60ies and 70ies, under from today’s demand view wrong boundary conditions. Sodium cooled fast reactors, ideally with metal fuel, were from reactor physical point of view the perfect choice to breed Plutonium in a highly efficient way. PUREX was the way to win the Pu to enlarge the nuclear armament of the cold war. In our view, the world has changed significantly and this requests defining the decisive criteria for the world of tomorrow. Based on this recognition we have to ask,”why should we build an advanced, cutting edge technology to solve a part of the problems of the historic and future nuclear power generation (spent fuel and long term activity/toxicity of actinides) following a development track defined by historic decisions taken under the historic boundary conditions? In our view, the development should be driven by the current or even better the anticipated future demand. We should define a new, innovative way to solve the problems of the future taking into account the current and the envisaged future demand. P&T is a part of a highly innovative future technology which is even more advanced than the Gen IV goals. The technological feasibility has been proven on laboratory scale using SFRs and processes developed from PUREX [8], but we are now obliged to find an optimal scheme for the whole fuel cycle if we want to be successful on industrial scale. The first step to develop a completely new, demand driven salt clean-up system is the detailed knowledge of the elements which disturb the long term sustainable operation of a molten salt fast reactor. Possible reasons can be in the reactor physics (negative influence on neutron economy), in the fuel chemistry (competition in the solubility with TRUs), in material issues (aggressive interaction with the structural material), or in the reactor safety (reducing the source term in accident cases or the decay heat production) [12].

Applying the basic requests for a wide spread sustainable nuclear power production in the future, the upcoming next level S-curve should focus on the ultimate, universal vision for all kinds of electric energy production which is, by the way, independent of the observed ‘energy production’ or more scientific energy conversion system itself. This general vision is much more advanced and broader than the development goals of the first reactors and even more advanced than the GEN-IV goals. The ultimate, universal vision for ‘energy production’ can be given with one simple, old fashioned historic phrase–‘perpetuum mobile’. It is consensus, amongst engineers and physicists, there are the laws of thermodynamics which prevent this vision from operating. However, it is a clear vision and provides a destination to drive research and innovation to the right point, into developing a reactor with the requested features: breed and burn its own fuel, limiting the waste production as much as possible. The ideal solution will be to come as close as possible to this vision, thus the vision will form the essential basis for a successful wide spread application of nuclear reactors as carbon free energy source. The key words for a mission developed out of the vision are:

- As little fresh resources as possible requested
- As few fresh waste as possible produced
- Highly economic, reliable, and secure
- Safe [5]

In an ideal configuration, this reactor can fulfil the requests of P&T as side effect–thus there will be no specific reactor design and fuel cycle development especially dedicated for P&T requested. This is in strong contrast to the currently propagated solutions [1]. Developing a reactor following the above mission will lead to a system providing the key for a long term success of nuclear power reactors to become a reliable major carbon free, sustainable ‘energy source’ for the future. Obviously, a reactor can’t be operated completely without resources like it would be requested by the vision but it would be a smart option to better use already existing resources which we currently consider as waste to fulfil the mission. This could be the spent nuclear fuel from existing light water reactors, however, without prior reprocessing like it is requested by all nuclear systems proposed in GenIV group. Finally, such a reactor wouldn’t require **fresh resources**, when operated on the basis of spent nuclear fuel (SNF) which exists in vast amounts as relict of the operation of the current reactors fleet. In addition, it would not produce **additional waste** since SNF is already considered as waste. However, this waste would be used to fuel the reactor to produce additional energy out of the given waste amount. The ideal design should also make misuse of plutonium as unattractive as possible, e. g. by avoiding the separation of Pu; it has to be reliable, safe, and economically competitive compared to any other source of electricity generation. If this can be achieved, the operation of a reactor based on SNF could even excel the objectives of the currently existing P&T strategies. The solution for the nuclear waste problem would be assured by using the electricity production system itself without producing additional long term wastes based on transuranium isotopes. In this case P&T does not have to be performed in special dedicated reactors if we are able to fulfil the requests directly in the system dedicated to electric energy generation. This will avoid the request for the development of dedicated burner reactors as well as the development of a special fuel cycle and the expected multi recycling scheme. However, this requests the design of an advanced fuel cycle or reprocessing scheme which is shaped to fulfil the demand of the reactor–separate the materials which prevent the rector from long term operation.

The sustainability of operation of a reactor based on already used LWR fuels is a hard challenge which has to be solved within the reactor core physics of a proposed reactor and an advanced fuel cycle scheme has to be built around. The primary challenge for this kind of nuclear system is to provide enough excess neutrons in the core to allow a self-sustained long term operation on the basis of SNF. Given this, the first request is to design a fast system which can produce the required amount of excess neutrons and to prove the operability of the proposed system from neutronics point of view. The below provided feasibility study is based on a molten salt fast reactor configuration close to proposed design of the EVOL project [13]. However, the configuration is only a proposal which needs further exploration and optimisation to realise the requested goals to the best possible extent. The potential advantageous safety behaviour and the excellent operational flexibility of molten salt reactors have already been extensively discussed [14, 15, 16, 17]. The challenges in the development of a fast molten salt reactor have already been described in several projects [5, 10, 16]. A figure describing the major R&D challenges is given in the discussion.

## Materials & methods

The raw data of the calculations (Aurora, HELIOS and ZENITH files), the used PYTHON script for the cycle calculations and the Excel file for the data acquisition are available under [18].

### Reference configuration

**Fig 2:**
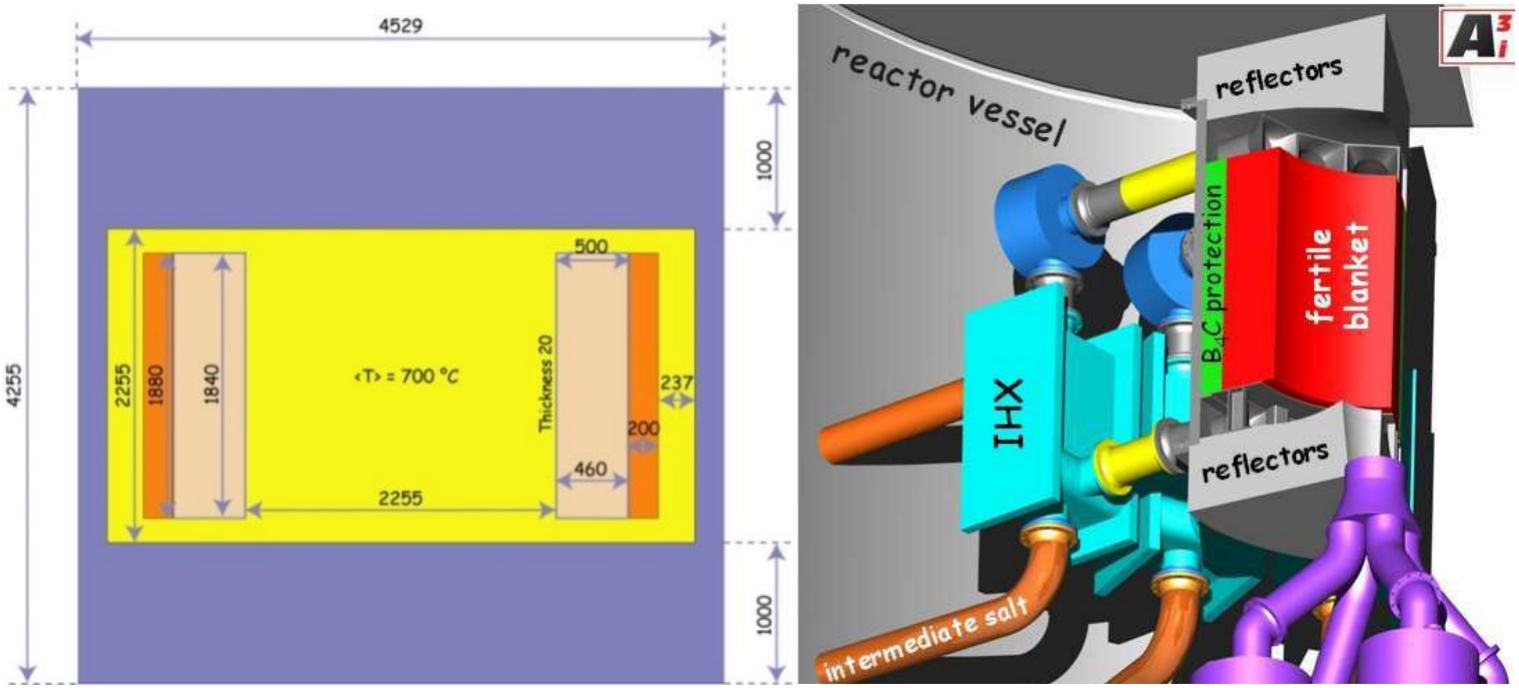
(Right): Simplified scheme of the MSFR system including the core, blanket and heat exchangers (IHX)–(Left): Benchmark definition [13].

The calculations are based on the core dimensions and boundary conditions given in the EVOL benchmark definition (see Fig 2) for a 3000 MW_th_ reactor system. The core is a single cylinder where the power production occurs, with the fuel salt flowing through the cylinder [18] providing the fuel function as well as the heat transport function. The dimensions are shown in Fig 2. The core is surrounded by a reflector and a protection ring, supposed to protect sensitive components from the high neutron flux arising within the inner core.

The spent nuclear fuel composition is calculated based on the “OECD/NEA AND U.S. NRC PWR MOX/UO2 CORE TRANSIENT BENCHMARK” [19] for 4.5% enriched Uranium oxide fuel. The detailed end of life fuel composition resulting from the HELIOS 2.1 calculation is given in table A of S1 File. To simulate storage time, the Pu-241 content has been halved and turned to Am-241, representing a postulated storage of 14 years. Besides this change no further adaption for storage has been introduced to the isotopic content. For the addition of fissile material for reactor start-up the transuranium (TRU)isotopic vector defined in the EVOL benchmark is used as well as the basic salt configuration consisting of LiF (18 m^3^) with mainly UF_4_ (applied as SNFF_4_ as approximation) in the core. The SNF share has been determined in a so-called material search, to achieve criticality of the system as well as sufficient breeding of fissile material to keep the reactor critical for long term operation without further feeding of separated fissile material. The power of the salt clean up system has to be adapted for the optimization the neutron economy and thus for the breeding. The blanket region is filled with pure LiF (7.7 m^3^) to be seen as a surrogate for a neutron physically inactive blanket salt in contrast to the fertile salt used in the EVOL benchmark composition.

### Modelling and simulation tool

**Fig 3:**
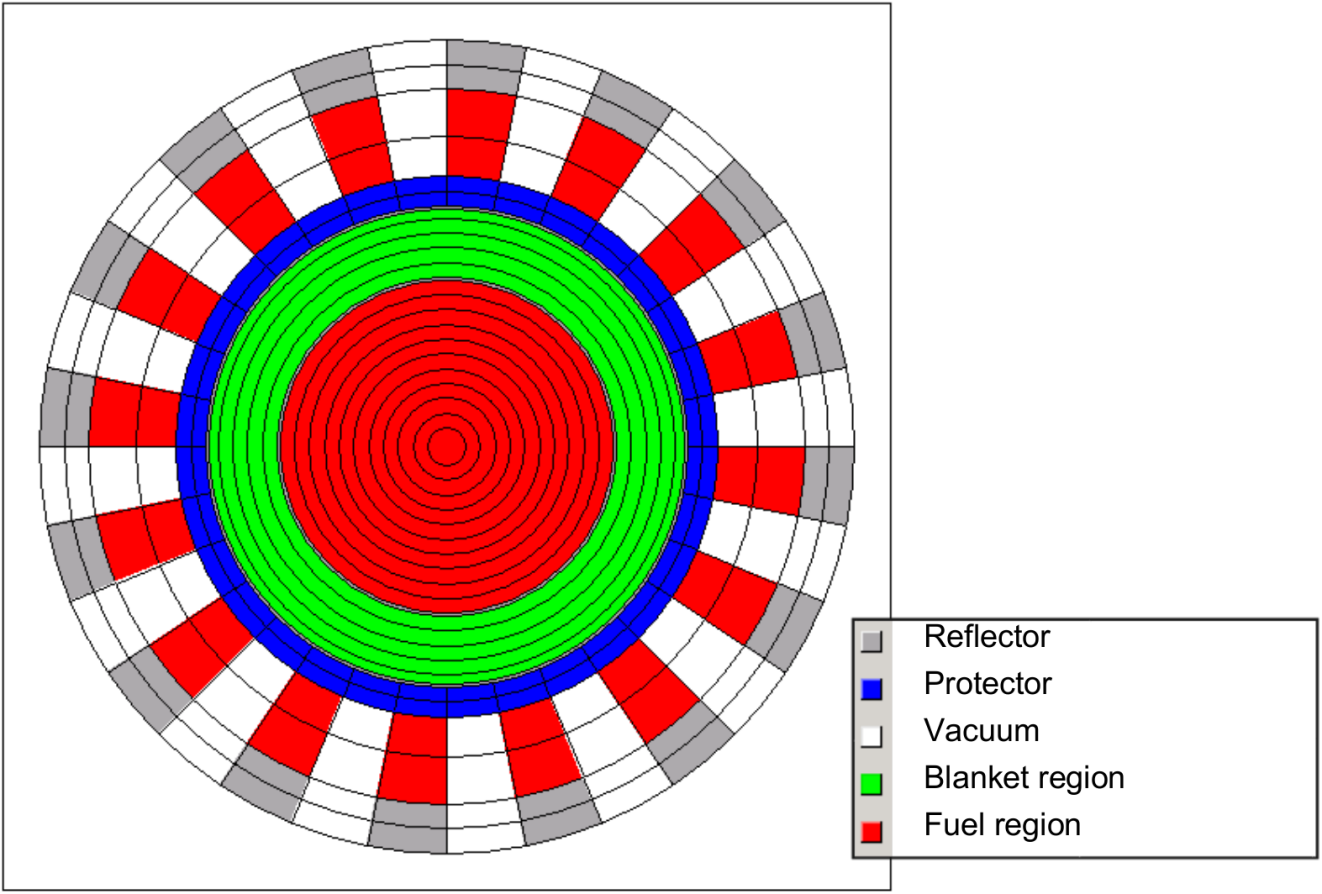
Volume corrected 2D HELIOS model of the molten salt reactor.

For the study, the licensing grade code system HELIOS 2.1 is used [20]. The code is a 2D spectral code with wide unstructured mesh capabilities and a transport solver, based on the collision probability method [21] and burnup capabilities. The benchmark configuration is transferred to a volume corrected 2D HELIOS model (see Fig 3) with vacuum boundary conditions and leakage in the third dimension based the on the EVOL benchmark exercises. The model has been refined using 16 heat exchanger pipes instead of the smeared treatment in the benchmark configuration to improve the neutron leakage approximation.

**Fig 4:**
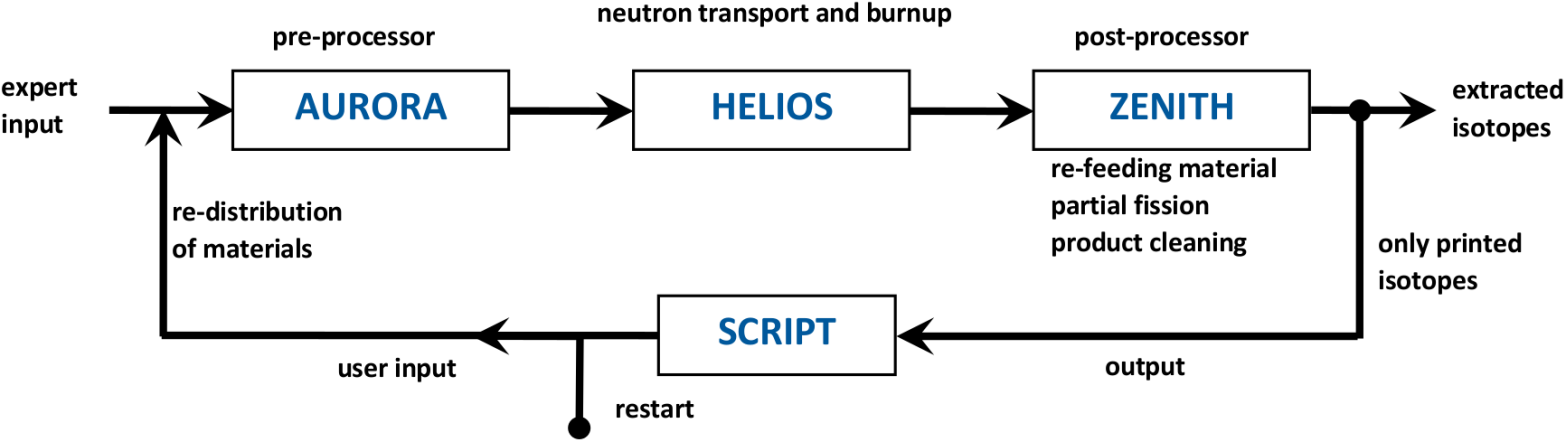
Description of the calculation cycle for the simulation of a MSR.

The HELIOS code is an industrial standard software for neutron transport and fuel burn up calculations. Originally, HELIOS was written for the simulation of solid structured fuel assemblies as used for the determination of the SNF configuration. Neither online refuelling nor online salt clean-up is foreseen. To deal with these processes a PYTHON script has been developed, based on the features of the HELIOS package. All information, which is constant during reactor operation is provided in the expert input, only the changing material configuration is given in the user input which is written new in every cycle using the PYTHON script. Within a cycle 5 burnup steps are calculated in HELIOS up to the target burnup of 5000 MWd/tHM. Both inputs are merged in the pre-processor AURORA to prepare the next HELIOS run. The results are evaluated for each cycle in the post-processor ZENITH, where the isotopes which remain in the salt and added SNF will be fed back into the next user input, created with the help of the PYTHON script (see Fig 4). This scheme could simulate a molten salt reactor precisely by using small time steps. However, in a real molten salt reactor two different time scales for the salt clean-up appear, one due to the helium bubbling for gaseous and volatile fission products with a short half-life time, and the online salt clean-up for dissolved fission products with a significantly longer half-life. To simulate this, a full removal of all gaseous and volatile fission products takes place after each cycle while only a partial removal of dissolved fission products is established.

Due to the characteristics of HELIOS being written for solid fuel simulation, some approximations have to be accepted, for example, there is no fuel salt movement, and thus a burnup distribution arises during one calculation cycle. The materials are only re-distributed when a new user input is defined via the script. HELIOS was designed for use with LWR reactors. However, comparisons to the SERPENT Monte Carlo code on the isotope accumulation during burnup in a fast reactor configuration have shown a good agreement for major isotopes [22]. However, the use of the HELIOS code package seems to be adequate for the level required for this kind of long term study where computational efficiency is essential. The major uncertainties are predominantly given by the current preliminary design. Such design uncertainties are expected to impact to a significantly higher extent on the results than the approximations made within the modelling and the code. In general, the calculations are performed to demonstrate the feasibility of the operation of a critical fast reactor on the basis of spent LWR fuel with high burnup.

## Results

### Initial core

The initial, critical core configuration averaged over the first cycle is based on ~70 tons of SNF without prior reprocessing and an initial seed of ~16 tons of TRU as fissile component for the start-up of the fast neutron system, which accumulates to 82 tons of HM. The values are in a comparable range to other kinds of nuclear reactors, e. g. ~86 tons of HM for a 1000 MWe LWR core [23], or ~130 tons of HM for a 1450 MWe LWR core, but are clearly higher than the 42 tons for a SFR core with 1450 MWe [24]. The detailed, initial isotopic composition of the molten salt is given in table A of S1 File.

### Simulation over lifetime

**Fig 5:**
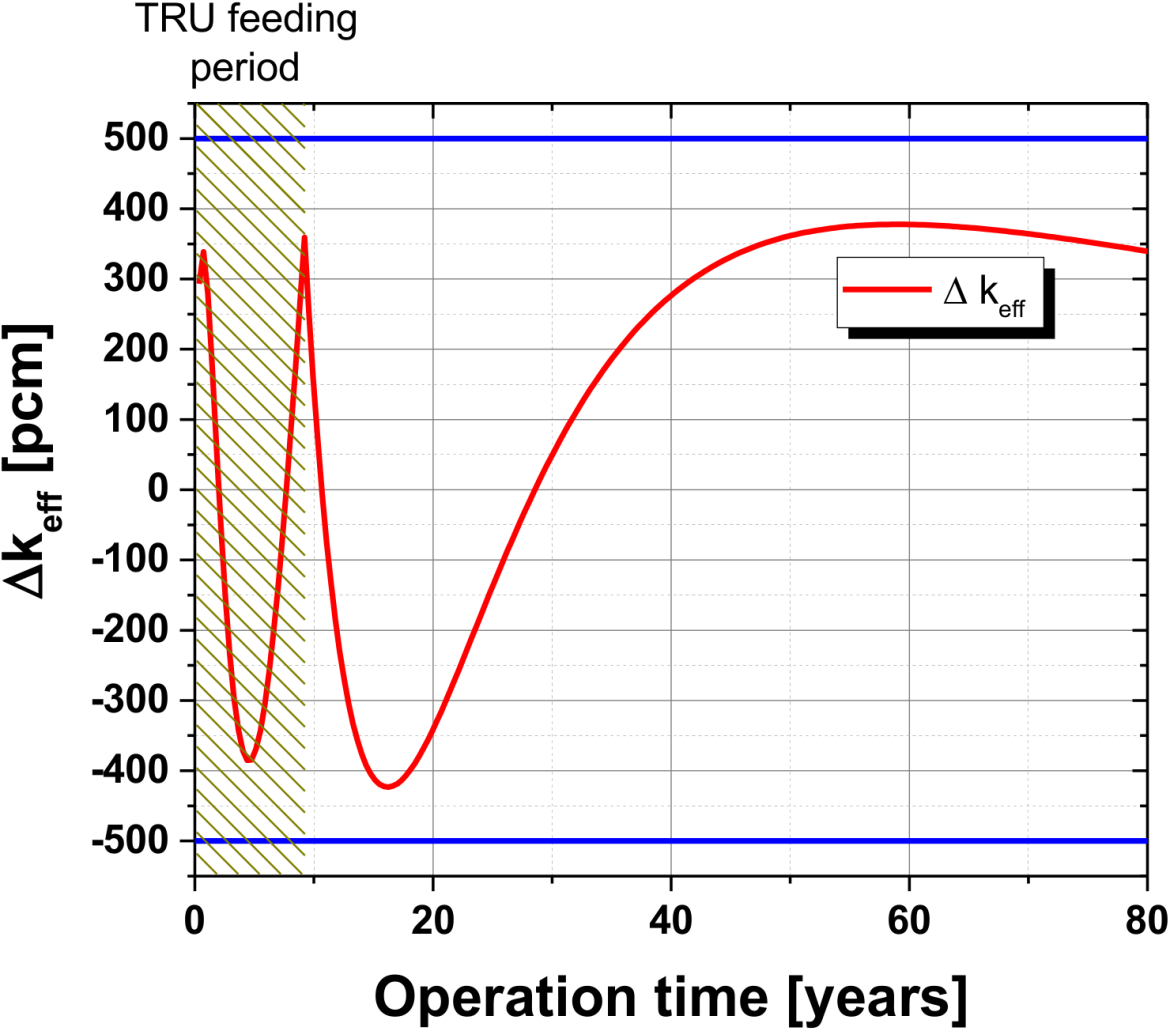
Change of the deviation of the averaged effective multiplication factor ${\bar{\Delta k}}_{\mathrm{eff}}$ over a simulated operational period of 80 years within the iteration band of ±400 pcm.

For the long term simulation a variable TRU feeding is used to keep the ${\bar{\Delta k}}_{\mathrm{eff}}$ in the iteration band of ~±400 pcm around critical, Fig 5. In the TRU feeding period over all ~1.8 tons of TRU are fed into the system. Additionally, a constant amount of SNF is fed into the system at each cycle to keep the U-238 level only slowly decreasing over the whole operational period. After the transformation of the isotopic composition to a fast reactor configuration the TRU feed is not needed anymore, since the system has become self-sustainable after less than years. Only SNF is fed into the system and the system criticality stays within the iteration band of ±400 pcm. Over the evaluated operational period of 60 years ~70 tons of SNF are fed into the system. The detailed, end of operation isotopic composition of the molten salt is given in table A of S2 File.

### Isotopic contents

The analysis of the isotopic content of actinides is one of the key parameters to be analysed for the investigation of the applicability of a reactor system for transmutation. The reactor system is only promising, when an accumulation of the higher isotopes can be avoided during operation. This is one of the key reasons why fast reactors are preferable for transmutation. The fission probabilities for most of the TRU isotopes are much higher in a fast reactor spectrum than in a thermal reactor spectrum [1, 10]. In this reactor concept, TRU isotopes are not only fed into the system during the TRU feeding period, but also through the SNF (which contains always a small amount of TRU which has been generated during LWR operation) which feed into the system. In an ideal, continuously fed transmutation system, an as low as possible asymptotic limit should be kept during operation while the feeding continues.

**Fig 6:**
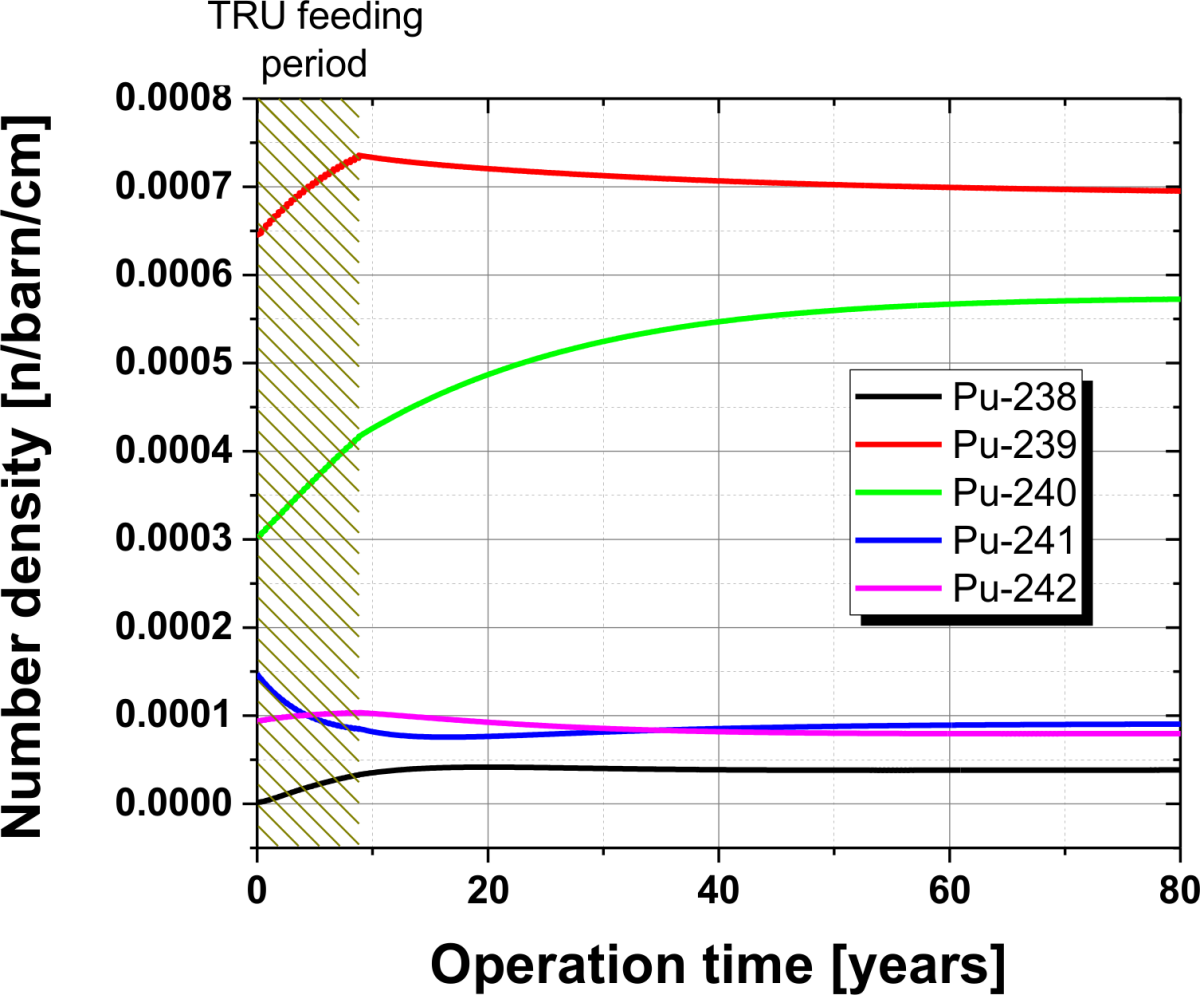
Number density of the different Pu isotope particles in the fuel salt over the observed operational period.

One of the major requests for the operation of a transmutation reactor is to avoid the continuous accumulation of Pu and other higher isotopes. The atomic number density of the most important fissile isotope Pu-239 increases during the first part of the TRU feeding period, as shown in Fig 6. This is an important point to keep the system critical which is a prerequisite for the self-sustained operation. From point of view of transmutation it is important to recognize that all Pu isotopes reach an asymptotic concentration during operation which obviously takes longest for Pu-240. The asymptotic behaviour is a basic request which has to be backed up by the right kind of separation process. A process is required which avoids the carryover of Pu into the waste stream when the lanthanides are separated.

**Fig 7:**
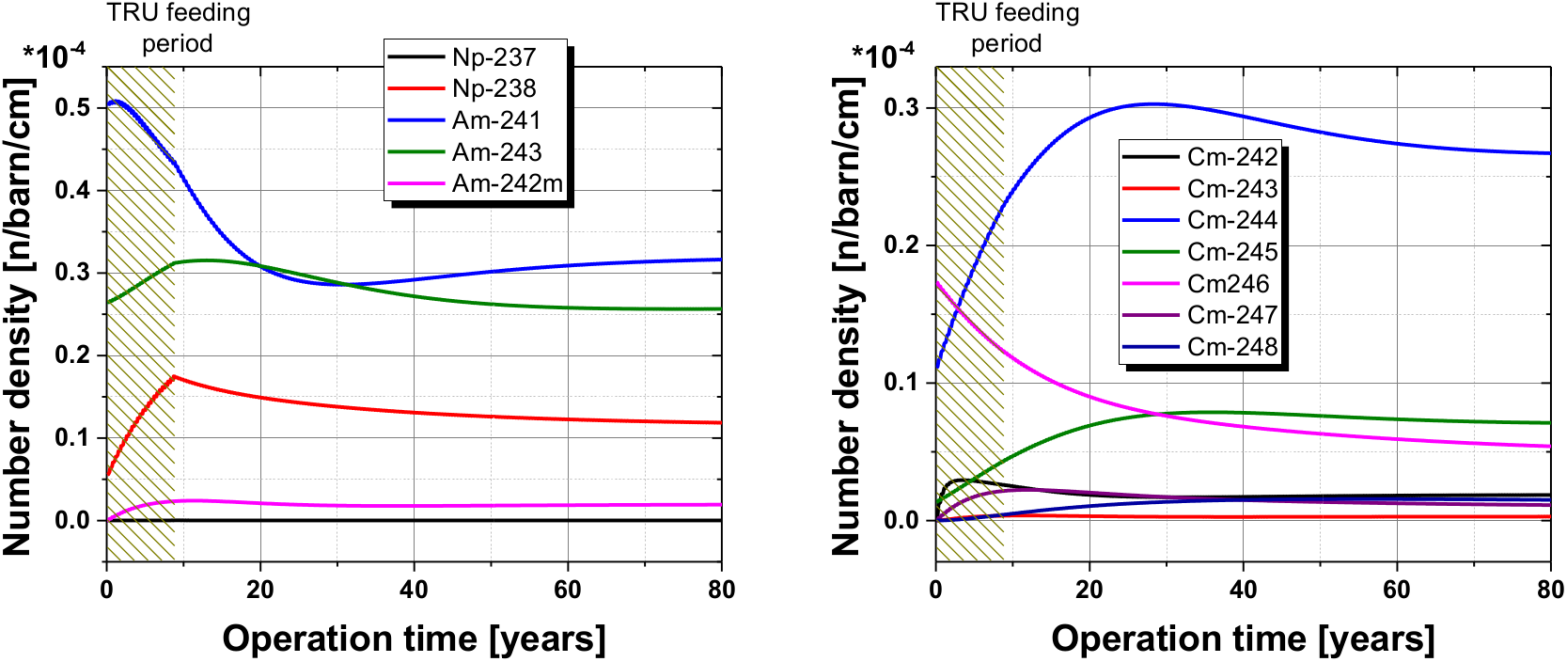
Number density of the relevant Neptunium and Americium (left) and Curium (right) isotope particles in the fuel salt over the observed operational period.

The detailed observation of the minor actinides over the operation time confirms the findings for the Pu isotopes. All Americium as well as Curium isotopes reach an asymptotic maximum concentration even if the isotopes are fed during the TRU feeding period as well as with the SNF, see Fig 7. The formation of Cm and the build-up of the maximum isotopic concentration takes longer due to the longer breeding chain (number of neutron capture reactions to form the observed isotope). However, after a maximum of 40 years all minor actinide isotopes have reached the maximum value.

Another important point of the isotopic analysis is the follow up of the Pu composition. This parameter is one of the key requests for the proliferation resistance of a reactor system. This has to be backed with two other requests. The first is the suppression of the possibility of a misuse of the reactor system by short term insertion and withdrawal of fertile material. This is in this reactor concept almost impossible since all inserted fertile material is liquid and would immediately be mixed with the bulk amount of salt. Thus, a misuse of the reflector or a massive intervention into the hardware would be required for unlawful Pu production. The second is once more in the design of the separation process. Here, it is essential that the Pu is not separated during the salt clean-up process to avoid any possibility of withdrawal of any Pu, even a less attractive Pu vector with high amounts of even Pu isotopes. It has already been discussed in the past by Engel et al, that this is possible in a MSR. Once the plutonium, or other fissile material, is put into a molten salt reactor system it is not necessary to separate the material, in order to the keep the fission process going [25]. The Pu compositions at the end of operation shows a dramatic change, when compared to the initial TRU feed, see Table 1. The share of LWR fissile Pu isotopes is decreasing from the already low content of TRU, itself taken from high burnup LWR fuel. The primary cause is significant build-up of Pu-240, see Fig 6, with the observed decrease of Pu-241 and 242, which is itself typical for the operation of a nuclear reactor with a fast neutron spectrum, where the accumulation of higher isotopes is strongly reduced compared to LWR with their immanent thermal neutron spectrum.

**Table 1:** The plutonium vector appearing in the reactor after long term operation compared to the Pu vector of the TRU used for loading.

Isotope	After 60 years	Load
Pu-238	3%	3%
Pu-239	47%	52%
Pu-240	38%	25%
Pu-241	6%	12%
Pu-242	5%	8%
Pu_fiss_	53%	64%

## Discussion

The results presented here demonstrate that a molten salt reactor with fast neutron spectrum, such as that proposed in the EVOL project, could be operated using pure spent nuclear fuel (SNF) from light water reactors after the transformation period has passed. In this chapter the wider consequences will be worked out.

The initial core configuration, as outlined above, within the model, 65% mol LiF, ~28.5% mol SNF, and ~6.5% mol TRU, translates to ~70 tons SNF and 16 tons TRU which in turn accumulate to ~82 tons of heavy metal (HM) while the blanket is filled with pure LiF salt as surrogate. A comparison with data of the original EVOL benchmark configuration core: 77.5%mol LiF and 22.5%mol HM (~16% Th and ~6.5% TRU), blanket: 77.5%mol LiF and 22.5%mol Th, indicates an increase of the actinide content. The full EVOL reactor (core and blanket) contains ~62 tons of HM compared to 74 tons of HM in the SNF core.

Over the TRU feeding period ~1 tons of TRU are fed into the system, whereas in the evaluated operational period of 60 years ~72 tons of SNF are fed into the system. Therefore, ~ 69 tons of heavy metal are fed, during an operation time of 60 years. A quick calculation for the burnt HM mass leads to a theoretical value of 66 tons, with a burning rate of 42 kg/TWh which is characteristic for a fertile free system where no fissile isotopes leave the reactor [16]. The salt clean-up system has to be enhanced compared to the EVOL benchmark. In the simulation here the 18m^3^ of molten salt is cleaned within two cycles, corresponding to a time ~250 days, depending on the HM present in the core which itself is changing. When leaving the level of the feasibility study to develop a possible reactor design, the salt compositions will one of the major optimization parameters. The key parameters for the choice of the salt will be the melting temperature, the required solubility [16] for TRU isotopes and fission products, the compatibility with the construction material, and the choice of the chemical processes for the salt clean-up [12].

**Fig 8:**
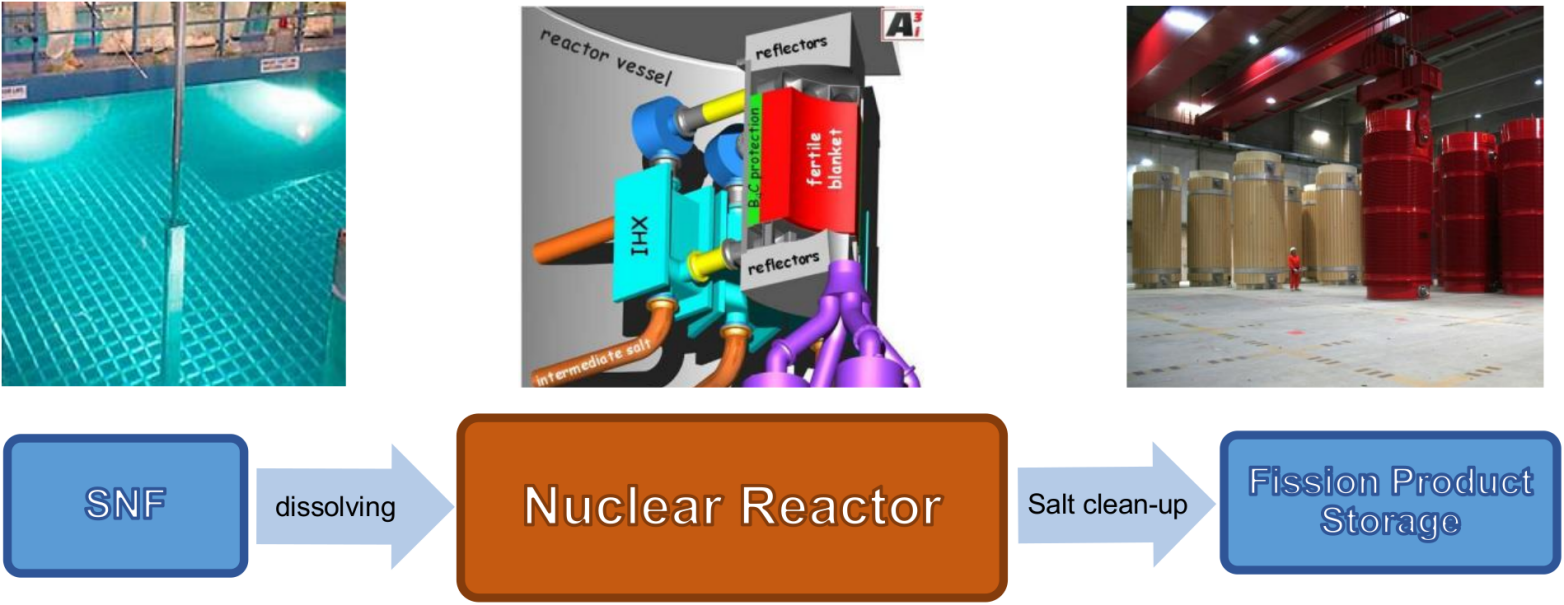
The reduced fuel cycle like it would be given using MSFR fed with SNF. Pictures: left http://www.wikiwand.com/en/Spent_fuel_pool, credit DOE License: Public domain, middle: EVOL benchmark configuration, right: Gorleben storage hall, Origin: GNS Gesellschaft für Nuklear-Service mbH.

Reactor operation based on SNF will lead to a significantly reduced fuel cycle (see Fig 8), when compared to current nuclear reactor operation scheme or the currently discussed P&T scheme. The fuel cycle for this kind of reactor operation would consist of the dissolution of the SNF to produce the fuel salt, the reactor operation itself, and the salt clean-up system. This has the potential to significantly reduce the environmental impact of nuclear power production since no new, fresh resources are required and thus no mining is required which itself causes significant toxicity; “Clearly, mining is the only contributor with more than 99% of the potential impact both for the eco and the human toxicity”[26].

**Fig 9:**
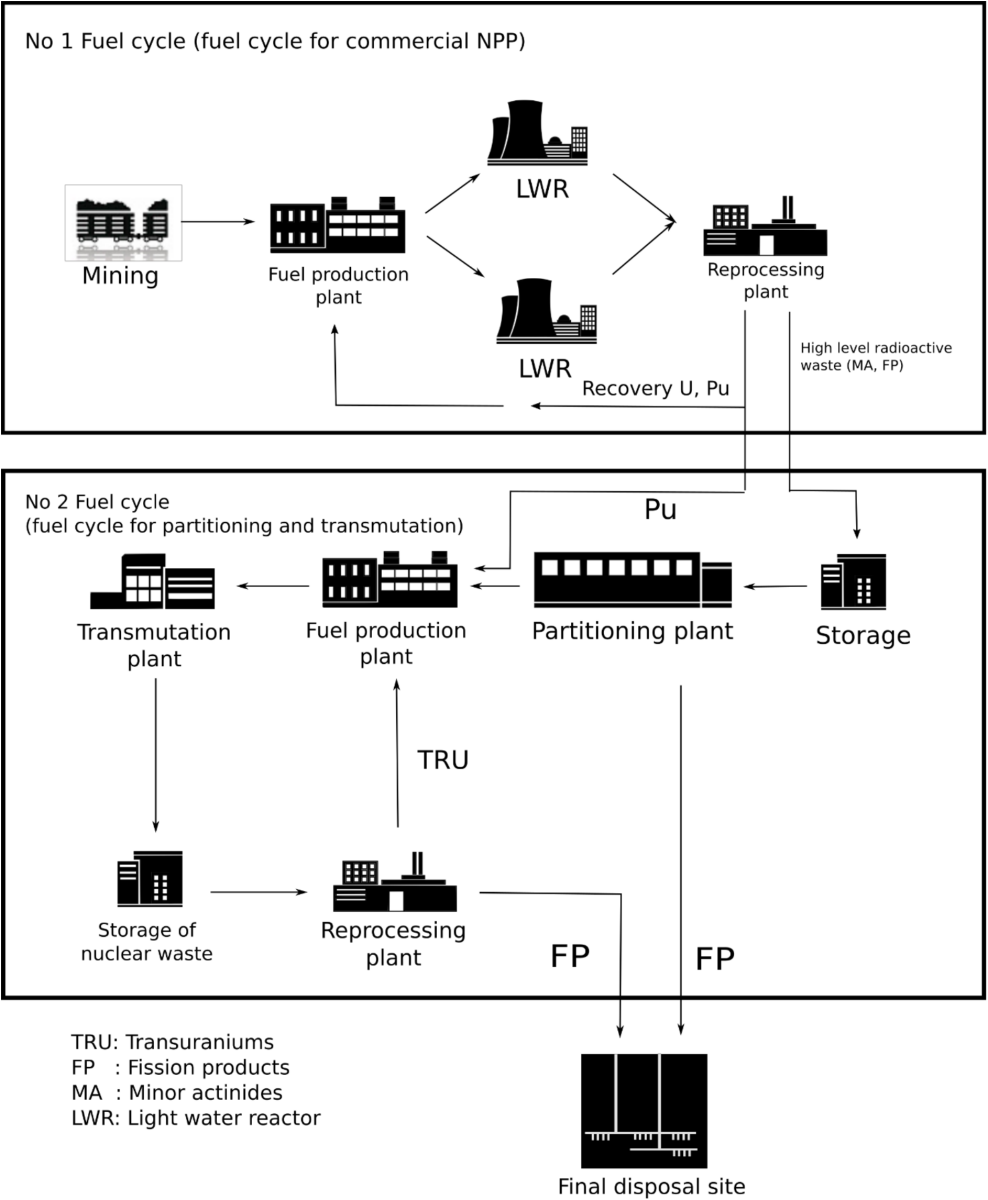
The classical double strata fuel cycle like it is foreseen for the implementation of P&T on industrial scale, today.

A comparison with the currently anticipated double strata fuel cycle of P&T (see Fig 9) demonstrates the extent of the reduction of the fuel cycle. Instead of different reactor types for power production and transmutation, different separation facilities for LWR fuels, minor actinides separation, and fast reactor fuel reprocessing only one co-located system is required. The components for storage of the fast reactor fuel are omitted completely and the challenge of fuel production is significantly reduced. In general, the closed fuel cycle option is not unique for molten salt reactors; any kind of fast reactor with sufficient breeding can be applied for this purpose. However, the liquid fuelled reactors can be seen as ideal for this purpose due to the significantly reduced fuel cycle without solid fuel production, storage requests for cooling of the used fuel, transporting, and explicit reprocessing. A special advantage is that the spent nuclear fuel can be inserted into the reactor without prior re-processing; only the initial step of the re-processing, the dissolution of the solid fuel would be required. The possibility of a demand driven salt clean-up (take out what prevents the reactor from long term operation instead of separate the fissile material) as discussed in the paragraph ‘demand driven research’ and the optimization between the required salt purification requests and efficiency as well as the throughput the clean-up system opens completely new options for the development of really innovative solutions [5, 12]. This has to be seen as one of the major challenges of the development of an integrated nuclear system (reactor and all related parts of the fuel cycle). As result of the close coupling of reactor operation and salt clean-up, the development will require a close interaction between all involved disciplines (physics, chemistry, process modelling, material and design engineering).

The co-location of reactor, salt clean-up (reprocessing), and fuel production without further transport, avoids a ‘Plutonium economy’ like it is currently required for a closed fuel cycle which is operated via the separation of actinides. No risky handover of Pu at the different stages is required. Additionally, separation of fissile material during reprocessing which would open possibilities for misuse can be avoided.

The penultimate design parameter is a nuclear reactor which doesn’t produce additional waste. There are different parameters which can be used for this evaluation; the waste mass and the activity, which can be separated into the short and the long term activity/toxicity. The long term activity/toxicity is the major parameter which has to be evaluated with regards of P&T, but in our view the other two parameters are of high importance, too. Operating a reactor on SNF, such as the one proposed here, leads to no significant change in the waste mass. It will be almost identical to SNF which is introduced into the reactor. However, from this waste mass has been ~20 times more energy produced. The short term radioactivity of the waste is proportional to the amount of energy generated since the energy per fission is almost identical for all fissile actinides. This means, the activity of the waste is expected to be ~20 higher than of the inserted SNF. However, ~ 20 times more energy is produced. The long term toxicity/activity is still clearly reduced compared to the SNF since the major carriers of the long term activity/toxicity are the actinides and to the highest extent the TRUs. No TRUs are expected in the waste stream from the MSFR as far as the adaption of the salt clean-up to the demand is successful.

Nevertheless, it does not relieve the plant operators from developing a strategy for the handling and storage of the separated fission products from the clean-up system. In addition to this, a strategy for the capturing and handling of volatile fission products, evaporating from the salt during reactor operation has to be developed. Both challenges are well known in standard molten salt reactor development, with significant work already completed [14, 27, 28]. Such development work should take place in parallel, and develop the optimal mechanism for the salt purification, and clean-up, and remains a key challenge in designing the nuclear system.

**Fig 10:**
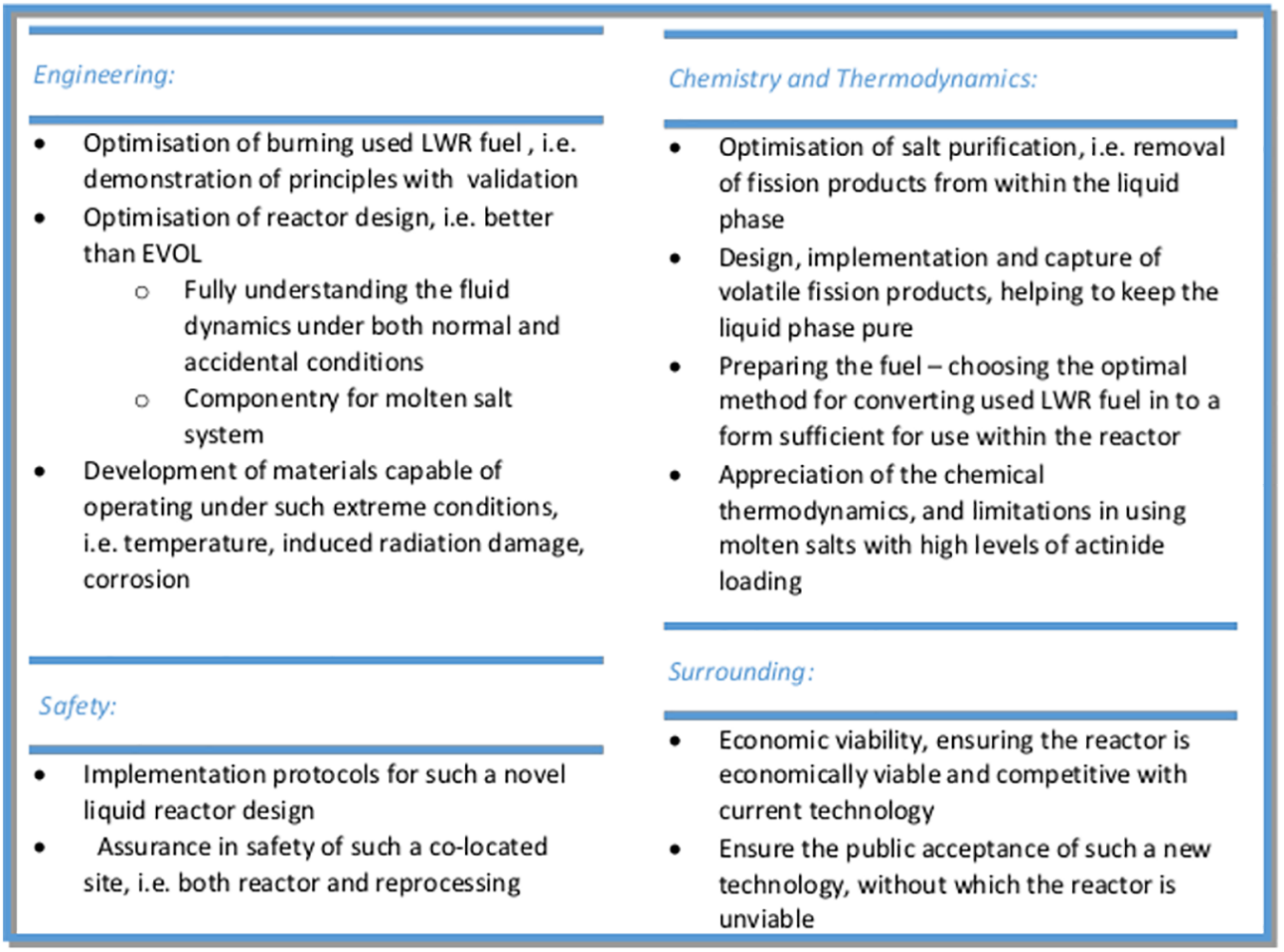
Overview on the major challenges for the development of a MSFR following [5].

The final requirement, i.e. the reactor to be safe, reliable and financeable, with limited financial risk in both development and construction, needs to be demonstrated, challenging engineers work. However, before starting such development work, and to assure that the system is working in the final design, it is essential to prove the reactor functionality regarding the operability on SNF and to confirm this at each important development decision. The major challenges on the way of a successful implementation of MSFRs for power production and P&T are given in *Fig 10*.

Besides the above challenges there remain other hurdles to overcome, as a consequence a step by step approach is foreseen developing operational experience, such that further progress can be made. Starting in a manner similar to the development of previous reactor systems, a small low power experimental machine will be required, which would lead to improved implementable designs. Such a machine has to be planned, built, and financed [16]. There is experience however, in the EVOL project, there have already proposals been made and published on solving the very high neutron fluences on safety related structures [29].

This tremendous research demand has to be recognized as one of the major challenges of establishing a completely new, demand driven technology (reactor as well as salt clean-up) which has to compete with much more mature technologies like the sodium cooled fast reactor technology with more than 400 reactor years of operational experience [8] and the industrially applied aqueous reprocessing technology. However, in our view the opportunities to establish a new technology which doesn’t require fresh resources, produces no additional waste masses and reduces the long term activity of the already existing waste justify to go new, disruptive ways in research to create the opportunities for a wide spread, highly efficient and reliable, low carbon energy source.

## Conclusions

The nuclear waste problem is recognized as the key problem for an extensive use of nuclear reactors as a major carbon free, sustainable, and applied highly reliable energy source. Partitioning and Transmutation (P&T) promises to provide a technological solution for improved nuclear waste management. However, the P&T technologies have only been demonstrated on laboratory scale up to now. Current strategies rely on traditional sodium cooled fast reactor systems and fuel cycle facilities which have been designed in the 60ies for the massive production of plutonium. We propose to support the technological development to the industrial application with a strategic development plan based on demand driven development. Invention and innovation in nuclear reactor development can be described with the concept of developments in s-curves. The identified key points which should drive invention and innovation are the changed boundary conditions, and the evolvable objectives. These new objectives ideally coincide with the ultimate, universal vision for energy production characterized by minimal use of resources and minimized production of waste, while being economically affordable and safe, secure, and reliable in operation. This vision can be transformed into a mission for the development of a future nuclear energy system based on liquid fuelled reactors which are operated on the basis of already existing spent nuclear fuel without prior re-processing. This approach is not only fulfilling the goals of sustainability in innovative electric energy production at the front end of the fuel cycle but has also the potential to create new solutions for the back end of the fuel cycle by fulfilling the requests of P&T.

A proof on the feasibility is provided based on neutronics point of view which provides a solution to the major challenge, establishing sufficient breeding of fissile material from the inserted spent nuclear fuel. For the initiation of the system a support of separated fissile material is required. Optimization of the design should be performed defining an ideal initial composition, and the required efficiency of the salt purification system to reduce the time required for the transition to a fast reactor configuration. It is demonstrated that a self-sustained system can be achieved and an accumulation of minor actinides can be avoided.

The result of such a design would be a system which does neither require new resources nor produce additional waste, providing an interesting, innovative option to sustainability of a future nuclear system. In addition to the desired sustainability, the request on an innovative waste management strategy based on P&T would be fulfilled in as an advantageous side effect. In addition, this nuclear system provides enhanced resistance against misuse of Pu due to the Pu composition, a very limited possibility for short time insertion fertile material and a design with Pu remaining in the system without further separations. The sustainability is supported by the significantly reduced environmental impact of the fuel cycle, which is reduced to 3 steps, i.e. dissolving the SNF, reactor operation, storage of fission products. This is a significant reduction of complexity compared to the currently discussed fuel cycle models required for P&T in traditional fast reactors. However, the new system requires a demand driven rethinking of the separation process to be efficient.

The design requirement of such an innovative system would be safe and financeable with limited financial risk in both development and construction which is a significant challenge. Even if the development of such a sustainable, and innovative nuclear reactor provides a major challenge across engineering, it opens the chance to provide a substantial impact on the future of the worldwide energy production.

## Supporting information

S1 File Spent LWR fuel composition. (DOCX)

S2 File Salt compositions. (DOCX)

## Abbreviations:

P&T: Partitioning and Transmutation
MSR: Molten Salt Reactor
MSFR: Molten Salt Fast Reactor
SNF: Spent Nuclear Fue
TRU: Transuranium–all elements with an atomic number higher than 92
HM: heavy metal–all elements with an atomic number higher than 90, typically the base to account for fissile materials in the reactor
MWth: Megawatt therma
GWe: Gigawatt electric
TWh: Terawatt hour
GWd/tHM: Gigawatt days per ton of heavy metal–measure for the burnup of nuclear material in a reactor
MWd/tHM: Megawatt days per ton of heavy metal
GenIV: Reactor systems of the 4th generation as defined by the Generation IV International Forum
$\Delta \bar{k}\mathrm{eff}$: difference from the critical reactor status, typically measured in pcm (percent mille
EoL: End of life
LiF: Lithium Fluoride
SNFF4: Spent Nuclear Fuel Tetrafluoride
UF4: Uranium Tetrafluoride
PUREX: Plutonium Uranium Redox EXtraction
GANEX: Group actinide extraction
COEX: Co-extraction, trademark of AREVA
SANEX: Selective ActiNide EXtraction
DIAMEX: DIAMideEXtraction
EVOL: European project for the development of a molten salt fast reactor
R&D: Research and Developmen
HELIOS: Reactor physics code package of Studsvik Scandpower
AURORA: pre-processor of the HELIOS package
ZENITH: post-processor of the HELIOS package
SERPENT: Reactor physics package based on the Monte Carlo method
ENDF/B VII: Nuclear data library
IAEA: International Atomic Energy Agency
