# Supplementary material for: An innovative way of thinking nuclear waste management – Neutron physics of a reactor directly operating on SNF
Source: PLoS One. 2017 Jul 27;12(7):e0180703. doi: 10.1371/journal.pone.0180703 (PMC5531547; doi:10.1371/journal.pone.0180703)
Supplement: S1 File — (DOCX) [file pone.0180703.s001.docx]

Table A: LWR spent fuel composition after a burnup of 50 GWd/tHM as calculated with HELIOS-2 based on the fuel of the NEA MOX benchmark

| isotopic identifier | LWR spent fuel composition (n/barn/cm) |
| --- | --- |
| 5010 | 1.28E-09 |
| 5011 | 1.15E-04 |
| 8001 | 4.53E-02 |
| 27059 | 1.00E-20 |
| 32576 | 3.80E-08 |
| 32577 | 1.80E-11 |
| 34577 | 8.66E-08 |
| 34578 | 2.41E-07 |
| 34579 | 4.72E-07 |
| 34580 | 1.33E-06 |
| 34582 | 3.30E-06 |
| 35581 | 2.11E-06 |
| 36582 | 6.83E-08 |
| 36583 | 3.80E-06 |
| 36584 | 1.08E-05 |
| 36585 | 2.22E-06 |
| 36586 | 1.74E-05 |
| 37585 | 9.39E-06 |
| 37586 | 2.50E-09 |
| 37587 | 2.26E-05 |
| 38587 | 2.08E-05 |
| 38588 | 3.29E-05 |
| 38589 | 1.70E-06 |
| 38590 | 4.76E-05 |
| 39589 | 3.92E-05 |
| 39590 | 1.25E-08 |
| 39591 | 2.63E-06 |
| 40000 | 7.17E-05 |
| 40590 | 2.42E-06 |
| 40591 | 4.96E-05 |
| 40592 | 5.63E-05 |
| 40593 | 6.13E-05 |
| 40594 | 6.55E-05 |
| 40595 | 4.19E-06 |
| 40596 | 6.74E-05 |
| 41595 | 2.30E-06 |
| 42595 | 5.70E-05 |
| 42596 | 3.93E-06 |
| 42597 | 6.64E-05 |
| 42598 | 6.82E-05 |
| 42599 | 2.08E-07 |
| 42600 | 7.53E-05 |
| 43599 | 6.13E-05 |
| 44600 | 1.05E-05 |
| 44601 | 6.13E-05 |
| 44602 | 6.38E-05 |
| 44603 | 2.75E-06 |
| 44604 | 4.28E-05 |
| 44605 | 9.51E-09 |
| 44606 | 1.09E-05 |
| 45103 | 1.00E-20 |
| 45603 | 3.14E-05 |
| 45605 | 6.97E-08 |
| 46604 | 2.03E-05 |
| 46605 | 2.97E-05 |
| 46606 | 1.74E-05 |
| 46607 | 1.65E-05 |
| 46608 | 1.09E-05 |
| 46610 | 3.64E-06 |
| 47107 | 1.00E-20 |
| 47109 | 1.00E-20 |
| 47609 | 5.62E-06 |
| 47611 | 2.38E-08 |
| 47710 | 7.90E-08 |
| 48110 | 1.00E-20 |
| 48111 | 1.00E-20 |
| 48112 | 1.00E-20 |
| 48113 | 1.00E-20 |
| 48114 | 1.00E-20 |
| 48610 | 2.89E-06 |
| 48611 | 1.80E-06 |
| 48612 | 9.76E-07 |
| 48613 | 7.37E-09 |
| 48614 | 1.20E-06 |
| 48616 | 4.48E-07 |
| 49113 | 1.00E-20 |
| 49115 | 1.00E-20 |
| 49615 | 1.35E-07 |
| 50616 | 3.13E-07 |
| 50617 | 4.05E-07 |
| 50618 | 4.04E-07 |
| 50619 | 3.87E-07 |
| 50620 | 3.93E-07 |
| 50622 | 4.76E-07 |
| 50623 | 7.77E-08 |
| 50624 | 7.18E-07 |
| 50625 | 1.16E-08 |
| 50626 | 1.43E-06 |
| 51621 | 3.94E-07 |
| 51623 | 3.88E-07 |
| 51624 | 3.64E-09 |
| 51625 | 6.02E-07 |
| 51626 | 1.11E-09 |
| 51627 | 1.69E-08 |
| 52622 | 3.47E-08 |
| 52623 | 3.97E-10 |
| 52624 | 2.07E-08 |
| 52625 | 2.84E-07 |
| 52626 | 6.21E-08 |
| 52628 | 5.85E-06 |
| 52630 | 2.37E-05 |
| 52632 | 1.88E-07 |
| 52727 | 1.10E-08 |
| 52729 | 6.07E-08 |
| 53627 | 3.01E-06 |
| 53629 | 9.42E-06 |
| 53630 | 6.17E-10 |
| 53631 | 3.37E-07 |
| 53635 | 2.19E-08 |
| 54628 | 2.50E-07 |
| 54629 | 2.04E-09 |
| 54630 | 4.57E-07 |
| 54631 | 2.42E-05 |
| 54632 | 6.83E-05 |
| 54633 | 4.42E-07 |
| 54634 | 9.14E-05 |
| 54635 | 7.94E-09 |
| 54636 | 1.34E-04 |
| 55633 | 6.60E-05 |
| 55634 | 8.26E-06 |
| 55635 | 2.44E-05 |
| 55636 | 3.74E-08 |
| 55637 | 7.11E-05 |
| 56634 | 3.66E-06 |
| 56635 | 3.10E-08 |
| 56636 | 1.31E-06 |
| 56637 | 3.07E-06 |
| 56638 | 7.61E-05 |
| 56640 | 8.94E-07 |
| 57639 | 7.06E-05 |
| 57640 | 1.23E-07 |
| 58640 | 6.91E-05 |
| 58641 | 2.15E-06 |
| 58642 | 6.38E-05 |
| 58643 | 8.30E-08 |
| 58644 | 1.57E-05 |
| 59641 | 6.14E-05 |
| 59642 | 2.81E-09 |
| 59643 | 8.21E-07 |
| 60642 | 1.38E-06 |
| 60643 | 4.14E-05 |
| 60644 | 6.05E-05 |
| 60645 | 3.58E-05 |
| 60646 | 3.99E-05 |
| 60647 | 2.90E-07 |
| 60648 | 2.01E-05 |
| 60650 | 9.52E-06 |
| 61647 | 8.06E-06 |
| 61648 | 4.50E-08 |
| 61649 | 5.29E-08 |
| 61651 | 1.00E-08 |
| 61748 | 7.50E-08 |
| 62152 | 1.00E-20 |
| 62153 | 1.00E-20 |
| 62154 | 1.00E-20 |
| 62647 | 4.10E-06 |
| 62648 | 7.94E-06 |
| 62649 | 9.64E-08 |
| 62650 | 1.50E-05 |
| 62651 | 6.15E-07 |
| 62652 | 5.50E-06 |
| 62653 | 4.66E-08 |
| 62654 | 1.95E-06 |
| 63151 | 1.00E-20 |
| 63152 | 1.00E-20 |
| 63153 | 1.00E-20 |
| 63154 | 1.00E-20 |
| 63155 | 1.00E-20 |
| 63156 | 1.00E-20 |
| 63157 | 1.00E-20 |
| 63651 | 6.46E-10 |
| 63652 | 4.63E-10 |
| 63653 | 6.41E-06 |
| 63654 | 1.44E-06 |
| 63655 | 4.48E-07 |
| 63656 | 2.25E-07 |
| 63657 | 8.00E-10 |
| 64152 | 1.00E-20 |
| 64153 | 1.00E-20 |
| 64154 | 1.00E-20 |
| 64155 | 1.00E-20 |
| 64156 | 1.00E-20 |
| 64157 | 1.00E-20 |
| 64158 | 1.00E-20 |
| 64160 | 1.00E-20 |
| 64652 | 1.72E-09 |
| 64653 | 1.38E-10 |
| 64654 | 1.43E-07 |
| 64655 | 3.49E-09 |
| 64656 | 5.00E-06 |
| 64657 | 5.15E-09 |
| 64658 | 1.01E-06 |
| 64660 | 5.57E-08 |
| 65159 | 1.00E-20 |
| 65160 | 1.00E-20 |
| 65161 | 1.00E-20 |
| 65659 | 1.27E-07 |
| 65660 | 3.66E-09 |
| 65661 | 4.60E-10 |
| 66160 | 1.00E-20 |
| 66161 | 1.00E-20 |
| 66162 | 1.00E-20 |
| 66163 | 1.00E-20 |
| 66164 | 1.00E-20 |
| 66660 | 1.12E-08 |
| 66661 | 1.83E-08 |
| 66662 | 1.39E-08 |
| 66663 | 1.04E-08 |
| 66664 | 2.68E-09 |
| 67165 | 1.00E-20 |
| 67665 | 4.89E-09 |
| 68162 | 1.00E-20 |
| 68164 | 1.00E-20 |
| 68166 | 1.00E-20 |
| 68167 | 1.00E-20 |
| 68168 | 1.00E-20 |
| 68170 | 1.00E-20 |
| 69169 | 1.00E-20 |
| 69170 | 1.00E-20 |
| 69171 | 1.00E-20 |
| 71175 | 1.00E-20 |
| 71176 | 1.00E-20 |
| 72174 | 1.00E-20 |
| 72175 | 1.00E-20 |
| 72176 | 1.00E-20 |
| 72177 | 1.00E-20 |
| 72178 | 1.00E-20 |
| 72179 | 1.00E-20 |
| 72180 | 1.00E-20 |
| 73181 | 1.00E-20 |
| 73182 | 1.00E-20 |
| 77191 | 1.00E-20 |
| 77193 | 1.00E-20 |
| 90230 | 3.84E-13 |
| 90232 | 9.11E-12 |
| 91231 | 7.82E-13 |
| 91232 | 2.30E-15 |
| 91233 | 5.43E-13 |
| 92232 | 1.24E-12 |
| 92233 | 3.17E-11 |
| 92234 | 1.03E-07 |
| 92235 | 2.00E-04 |
| 92236 | 1.39E-04 |
| 92237 | 2.88E-07 |
| 92238 | 2.09E-02 |
| 93236 | 7.61E-11 |
| 93237 | 1.58E-05 |
| 93238 | 4.83E-08 |
| 93239 | 2.07E-06 |
| 94236 | 1.93E-12 |
| 94237 | 1.74E-11 |
| 94238 | 6.58E-06 |
| 94239 | 1.33E-04 |
| 94240 | 6.16E-05 |
| 94241 | 3.84E-05 |
| 94242 | 1.82E-05 |
| 94243 | 4.94E-09 |
| 95241 | 1.32E-06 |
| 95242 | 3.50E-09 |
| 95243 | 4.47E-06 |
| 95342 | 2.07E-08 |
| 96242 | 5.21E-07 |
| 96243 | 1.37E-08 |
| 96244 | 1.80E-06 |
| 96245 | 1.25E-07 |
| 96246 | 1.42E-08 |
| 96247 | 1.97E-10 |
| 96248 | 1.47E-11 |
| 97249 | 1.52E-13 |
| 98249 | 2.28E-14 |
| 98250 | 7.12E-14 |
| 98251 | 3.07E-14 |
| 98252 | 1.99E-14 |
