# Supplementary material for: An innovative way of thinking nuclear waste management – Neutron physics of a reactor directly operating on SNF
Source: PLoS One. 2017 Jul 27;12(7):e0180703. doi: 10.1371/journal.pone.0180703 (PMC5531547; doi:10.1371/journal.pone.0180703)
Supplement: S2 File — (DOCX) [file pone.0180703.s002.docx]

Table A: Observed isotopic content of soluble isotopes following the EVOL benchmark isotope list for the initial configuration and the final configuration.

| isotopic identifier | initial number density (n/barn/cm) | final number density (n/barn/cm) |
| --- | --- | --- |
| 3006 | 1.20E-07 | 1.20E-07 |
| 3007 | 1.20E-02 | 1.20E-02 |
| 9019 | 3.95E-02 | 3.95E-02 |
| 35581 | 2.83E-07 | 1.12E-07 |
| 38589 | 1.96E-07 | 3.33E-07 |
| 38590 | 6.19E-06 | 1.59E-06 |
| 39589 | 5.15E-06 | 9.92E-07 |
| 39591 | 3.20E-07 | 5.38E-07 |
| 40591 | 6.54E-06 | 1.35E-06 |
| 40593 | 8.16E-06 | 2.74E-06 |
| 40595 | 5.87E-07 | 1.05E-06 |
| 40596 | 9.11E-06 | 3.56E-06 |
| 48610 | 3.59E-07 | 2.61E-08 |
| 48611 | 2.78E-07 | 2.67E-07 |
| 48613 | 2.23E-08 | 1.06E-07 |
| 49615 | 2.74E-08 | 5.46E-08 |
| 53627 | 4.30E-07 | 2.93E-07 |
| 53629 | 1.34E-06 | 8.32E-07 |
| 53631 | 5.89E-08 | 1.24E-07 |
| 53635 | 3.69E-09 | 7.61E-09 |
| 55633 | 9.09E-06 | 4.48E-06 |
| 55634 | 9.90E-07 | 1.10E-07 |
| 55635 | 4.21E-06 | 5.05E-06 |
| 55636 | 6.46E-09 | 1.07E-08 |
| 55637 | 9.76E-06 | 4.62E-06 |
| 56634 | 5.55E-07 | 2.96E-08 |
| 56637 | 4.46E-07 | 6.48E-08 |
| 56640 | 1.44E-07 | 2.94E-07 |
| 57639 | 1.16E-05 | 5.18E-06 |
| 57640 | 2.29E-08 | 3.89E-08 |
| 58640 | 1.13E-05 | 4.54E-06 |
| 58641 | 4.04E-07 | 6.84E-07 |
| 58642 | 1.04E-05 | 4.43E-06 |
| 58643 | 1.53E-08 | 2.60E-08 |
| 58644 | 2.44E-06 | 2.16E-06 |
| 59641 | 9.96E-06 | 3.93E-06 |
| 59643 | 1.51E-07 | 2.56E-07 |
| 60642 | 2.39E-07 | 3.89E-08 |
| 60643 | 6.92E-06 | 3.62E-06 |
| 60644 | 9.64E-06 | 1.55E-06 |
| 60645 | 5.85E-06 | 2.72E-06 |
| 60646 | 6.47E-06 | 2.46E-06 |
| 60647 | 5.87E-08 | 9.88E-08 |
| 60648 | 3.31E-06 | 1.57E-06 |
| 60650 | 1.61E-06 | 9.29E-07 |
| 61647 | 1.45E-06 | 1.40E-06 |
| 61648 | 1.86E-09 | 2.01E-09 |
| 61649 | 7.55E-09 | 1.27E-08 |
| 61651 | 2.33E-09 | 4.03E-09 |
| 61748 | 1.18E-08 | 1.16E-08 |
| 62647 | 6.96E-07 | 2.37E-07 |
| 62648 | 1.23E-06 | 1.38E-07 |
| 62649 | 2.73E-07 | 9.64E-07 |
| 62650 | 2.21E-06 | 2.32E-07 |
| 62651 | 2.47E-07 | 5.66E-07 |
| 62652 | 9.38E-07 | 6.44E-07 |
| 62653 | 2.31E-09 | 3.73E-09 |
| 62654 | 3.41E-07 | 2.53E-07 |
| 63651 | 4.82E-10 | 2.36E-09 |
| 63653 | 9.82E-07 | 3.48E-07 |
| 63654 | 2.49E-07 | 5.00E-08 |
| 63655 | 1.14E-07 | 1.50E-07 |
| 63656 | 6.50E-09 | 1.03E-08 |
| 63657 | 1.37E-10 | 2.51E-10 |
| 64654 | 2.65E-08 | 2.99E-09 |
| 64655 | 4.98E-09 | 1.25E-08 |
| 64656 | 7.85E-07 | 1.41E-07 |
| 64657 | 2.76E-08 | 7.43E-08 |
| 64658 | 1.60E-07 | 6.08E-08 |
| 64660 | 1.08E-08 | 1.32E-08 |
| 65659 | 2.42E-08 | 2.42E-08 |
| 65660 | 7.93E-10 | 8.22E-10 |
| 65661 | 1.20E-10 | 2.50E-10 |
| 66660 | 2.33E-09 | 1.71E-09 |
| 66661 | 3.88E-09 | 6.37E-09 |
| 66662 | 2.66E-09 | 3.83E-09 |
| 66663 | 1.74E-09 | 1.69E-09 |
| 66664 | 5.40E-10 | 8.74E-10 |
| 67665 | 7.50E-10 | 3.68E-10 |
| 90230 | 1.18E-13 | 4.04E-10 |
| 90232 | 2.53E-12 | 2.81E-11 |
| 91231 | 2.00E-13 | 5.35E-11 |
| 91233 | 1.97E-13 | 5.11E-13 |
| 91234 | 1.00E-20 | 1.00E-20 |
| 92232 | 3.44E-13 | 1.37E-10 |
| 92233 | 9.18E-12 | 5.43E-10 |
| 92234 | 3.04E-08 | 5.82E-06 |
| 92235 | 4.68E-05 | 7.85E-06 |
| 92236 | 3.43E-05 | 3.09E-05 |
| 92237 | 4.22E-08 | 3.95E-08 |
| 92238 | 5.09E-03 | 5.05E-03 |
| 93237 | 6.33E-06 | 1.23E-05 |
| 93238 | 6.07E-09 | 1.24E-08 |
| 93239 | 8.57E-07 | 8.68E-07 |
| 94236 | 8.39E-13 | 6.62E-11 |
| 94238 | 2.37E-06 | 3.95E-05 |
| 94239 | 6.48E-04 | 7.08E-04 |
| 94240 | 3.07E-04 | 5.85E-04 |
| 94241 | 1.41E-04 | 9.26E-05 |
| 94242 | 9.46E-05 | 8.39E-05 |
| 95241 | 5.05E-05 | 3.18E-05 |
| 95242 | 1.47E-08 | 9.41E-09 |
| 95243 | 2.65E-05 | 2.72E-05 |
| 95342 | 2.13E-07 | 1.94E-06 |
| 96242 | 1.24E-06 | 1.89E-06 |
| 96243 | 1.62E-08 | 2.94E-07 |
| 96244 | 1.17E-05 | 2.93E-05 |
| 96245 | 1.47E-06 | 7.91E-06 |
| 96246 | 1.66E-05 | 6.23E-06 |
| 96247 | 1.93E-07 | 1.32E-06 |
| 96248 | 2.71E-09 | 1.60E-06 |
| 97249 | 1.22E-11 | 3.67E-08 |
| 98249 | 7.53E-13 | 1.48E-07 |
| 98250 | 2.70E-13 | 6.73E-08 |
| 98251 | 9.22E-15 | 2.23E-08 |
| 98252 | 4.47E-15 | 1.84E-09 |
